# PEDOT:PSS-Loaded Gelatin Cryogels for Electrically Controlled Drug Release

**DOI:** 10.1021/acsomega.5c02628

**Published:** 2025-07-17

**Authors:** Huai-An Chen, Ching-Yu Lee, Bo-Jun Huang, Kai-Yun Tu, Pei-Hsuan Sung, Le Ngoc Hoang, Ching-Li Tseng

**Affiliations:** 1 Graduate Institute of Biomedical Materials and Tissue Engineering, College of Biomedical Engineering, Taipei Medical University, Shuang-Ho Campus, No.301, Yuantong Road, Zhonghe District, New Taipei City 23564, Taiwan; 2 Department of Orthopedics, Taipei Medical University Hospital, No. 252, Wuxing Street, Xinyi District, Taipei City 11031, Taiwan; 3 Department of Orthopedics, School of Medicine, College of Medicine, 38032 Taipei Medical University, No. 250, Wuxing Street, Xinyi District, Taipei City 11031, Taiwan; 4 Taipei Municipal Zhongshan Girls High School, No. 141, Section 2, Changan East Road, Zhongshan District, Taipei City 10455, Taiwan; 5 International Ph.D. Program in Biomedical Engineering, College of Biomedical Engineering, Taipei Medical University, Shuang-Ho Campus, No.301, Yuantong Road, Zhonghe District, New Taipei City 23564, Taiwan; 6 Research Center of Biomedical Devices, College of Biomedical Engineering, 38032 Taipei Medical University, No. 250, Wuxing Street, Xinyi District, Taipei City 11031, Taiwan; 7 International Ph.D. Program in Cell Therapy and Regenerative Medicine, College of Medicine, 38032 Taipei Medical University, No. 250, Wuxing Street, Xinyi District, Taipei City 11031, Taiwan

## Abstract

Conductive cryogel is a new form of biomaterial that provides additional electrical properties, expanding its applications in biomedical engineering, particularly in tissue regeneration, such as neural and muscle tissues, and in drug-delivery systems. In this study, the gelatin cryogels (GCs) incorporating the conductive polymer PEDOT:PSS (PGC) at an optimal ratio (PGC2) could achieve suitable conductivity as an electrically responsive scaffold applied for controlled drug release. The characterization results demonstrated that PGC2 exhibited a well-defined porous structure (90–190 μm), a favorable water uptake capacity (2738.4%), degradability (26.1% degradation after 24 h), and reversible deformability upon compression, demonstrating its suitability for minimally invasive procedures via injection. The electron-transfer capability and higher conductivity of PGC2 were also confirmed by examining the electrochemical properties, which revealed a lower impedance (0.0174 MΩ) compared to nonconductive GC2 (0.0208 MΩ). Additionally, drug release tests revealed that drugs contained in PGC2 as electrically responsive scaffolds had a higher release response to electrical stimulation (ES) (23%) compared to the one without ES (7%). Cytocompatibility assays revealed that NIH-3T3 cells cocultured on PGC2 and those cultured with a PGC2 extraction solution exhibited cell viability above 70%, indicating nontoxicity, as confirmed by a WST-1 assay and a live/dead staining assay. Overall, PGC2 represents a promising biomaterial with injectability, conductivity, and cytocompatibility, offering potential applications in regenerative medicine, drug delivery, and bioelectronic scaffolding.

## Graphical abstract

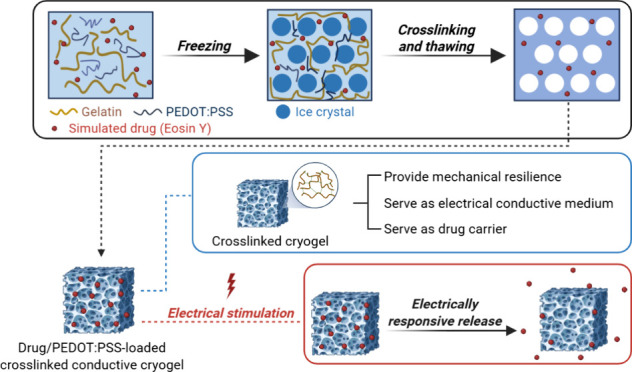

## 1 Introduction

An ideal biomimetic scaffold applied for tissue regeneration should possess some key characteristics, such as high porosity, interconnected networks, biocompatibility, biodegradability, and certain mechanical strengths.^1^ Three-dimensional (3D) polymeric network hydrogels have high water contents and, depending on their specific chemical structure and mechanics, can behave similarly to natural tissues; consequently, they have potential for use as tissue scaffolds.^2^ However, some nanoscale pores in hydrogels can restrict cellular ingrowth/migration and nutrient/oxygen diffusion, thereby affecting cell viability and function, motivating the development of macroporous gels to circumvent these problems.^3^

A new type of gel, polymer cryogels, with supermacroporous gel networks developed by the cryogelation of apposite monomers or polymeric precursors at subzero temperatures, is an emerging class of biomaterials that has garnered attention as potential scaffold preparations for applications in regenerative medicine.^4^ The macroporous structure of cryogels, formed by ice crystals, supports cell migration and proliferation, with interconnected pores that also provide enhanced elasticity, mechanical resilience, and permeability.^3,5^ These features make cryogels highly suitable for tissue regeneration and drug delivery. Gelatin-based cryogels (GC), due to the natural properties of gelatin, provide a biocompatible and biomimetic environment as an extracellular matrix (ECM) that supports cell adhesion, proliferation, and differentiation; they also provide improved mechanical properties and injectability for *in vivo* applications.^6^ These advantages make cryogels an ideal scaffold structure for applications in regenerative medicine and other biomedical fields.^6^

Bioelectrical signals can help the cytoskeleton rearrange and plasma membranes depolarize, alter the integrin receptor conformation, and modulate transmembrane calcium influx.^7^ Therefore, incorporating conductive materials to create electroconductive scaffolds can enhance the functionality of engineered scaffolds.^8,9^ For example, the addition of silver nanoparticles (Ag NPs) significantly improved conductivity while exhibiting excellent antibacterial properties, reducing the risk of infection, and promoting cell proliferation, making them highly valuable for wound-healing applications.^8,10^ Furthermore, integrating carbon nanotubes (CNTs) and graphene oxide (GO) into polymer matrices not only increased the material’s conductivity but also enhanced the mechanical strength and facilitated cell adhesion and proliferation, contributing to improved tissue regeneration.^8,10^ These materials collectively strengthen the structural and functional properties of scaffolds, enabling advanced applications such as electrically responsive drug release, modulation of cellular behavior through electrical stimulation, and the promotion of wound healing, ultimately expanding the therapeutic potential of tissue-engineered constructs in regenerative medicine. The electrical property can help, particularly in the repair and functional restoration of neural, muscle, and bone tissues.^8^ However, most carbon- and metal-based nanomaterials are insoluble in water, are prone to aggregation, and have potential cytotoxicity due to their size, structure, and surface properties,^10^ limiting their biological applications; therefore, alternative materials are being sought to replace them.

Conductive polymers, such as polypyrrole (PPy), polythiophene (PT), polyaniline (PANI), and poly­(3,4-ethylenedioxythiophene):poly­(styrenesulfonic acid) (PEDOT:PSS), are organic materials with conjugated π-orbitals that enable electron delocalization and high electrical conductivity. These polymers have recently emerged as promising candidates for applications in the biomedical field.^11,12^ Due to their excellent conductivity, processability, and biocompatibility, these conductive polymers have garnered considerable attention.^13^ Among them, PEDOT:PSS stands out for various advantages, including ease of processing, low costs for fabrication, water solubility, chemical and physical stability, excellent conductivity, and biocompatibility, making it the most promising conductive polymer for biomedical applications.^11,14^ Moreover, its conductivity can be easily tuned through various doping and processing methods, allowing for precise control for various applications, including bioelectronics, flexible devices, and tissue regeneration.^15^ A previous study demonstrated that PEDOT:PSS-based composites promoted enhanced differentiation, adhesion, and viability of MC3T3-E1 osteogenic precursor cells.^16^ The PEDOT:PSS was integrated into scaffolds to achieve electrical properties, then helped cells respond to electrical stimulation (ES), thereby promoting tissue regeneration and accelerating skin wound healing.^17^ The development of PEDOT:PSS-based materials holds great promise for tissue engineering studies, as it offers scaffolds that not only enhance electrical cues but also improve mechanical properties, ultimately facilitating therapeutic outcomes.

Beyond those advantages mentioned above for conductive biopolymeric scaffolds, conductive polymers are being explored for electrically responsive drug delivery, offering localized and tunable drug release to meet therapeutic demands.^18^ PEDOT:PSS was added to various hydrogels, preparing an electrically responsive drug-delivery system. For instance, PEDOT:PSS-loaded gelatin methacryloyl-based hydrogels showed an increase in 5-fluorouracil release from 18.2% in normal conditions to 23.3% using ES (1.5 V) due to electrostatic interactions causing hydrogel deswelling.^19^ Similarly, PEDOT:PSS-loaded alginate-based hydrogels released 15 times more curcumin under ES (−1.0 V), attributed to electrostatic repulsion-induced swelling.^20^ These results suggest that adding PEDOT:PSS into scaffolds can potentially serve as a drug-delivery platform responsive to ES.

Recently, studies have demonstrated the potential of cryogels in stimulus-responsive applications. Redox-responsive cryogels enabled protein immobilization and release via disulfide-thiol exchange, along with cell attachment and detachment.^21^ The photodegradable cryogels using poly­(2-oxazoline)-based cross-linkers were shown to degrade upon ultraviolet (UV) exposure.^22^ While PEDOT:PSS-loaded cryogels have been reported,^23^ those enabling both injectability and electrically responsive drug release have not yet been developed.

In this study, a gelatin-based cryogel (GC) was designed and prepared. Various parameters for synthesizing a suitable GC were studied. Thereafter, the conductive polymer, PEDOT:PSS, was loaded into the GC, which contributes to drug-release control via electrical stimulation; this PEDOT:PSS-containing GC is referred to here as PGC. The physical properties of the GC and PGC were examined, including the morphology, pore size, water uptake capacity, mechanical properties, injectability, degradation rate, and electrochemical properties. Then, the drug release rate was also examined with or without electrical stimulation. Furthermore, the in vitro cytocompatibility of these cryogels was also evaluated by cell viability and Live/Dead staining methods to assess their safety for application in the biomedical field.

## 2 Materials and Methods

### 2.1 Materials and Chemicals

Gelatin (type B, 225 g bloom number), glutaraldehyde (GA), PEDOT:PSS, and Eosin Y were purchased from Sigma-Aldrich (St. Louis, MO, USA). Phosphate-buffered saline (PBS) was purchased from BIOMAN Scientific (Taipei, Taiwan). Dulbecco’s Modified Eagle Medium/Nutrient Mixture F-12 (DMEM/F12) and trypsin-EDTA were acquired from Gibco (Grand Island, NY, USA). Fetal bovine serum (FBS) was purchased from NQBB International Biological (Hong Kong, China). Penicillin-streptomycin was obtained from Thermo Fisher Scientific (Waltham, MA, USA). A Cell Counting Kit-8 (CCK-8) was purchased from Dojindo Laboratories (Kumamoto, Japan). A Live/Dead Double Staining Kit was bought from Merck Millipore (Billerica, MA, USA).

### 2.2 Preparation of PEDOT:PSS-Loaded Gelatin Cryogels

Gelatin cryogels (GC) were prepared by cryogelation as described in the previous study with slight modification.^24^ First, gelatin underwent a desolvation process to narrow its molecular range; gelatin powder was added to warm deionized (DI) water (40 °C) for dissolution, and then an excess amount of acetone was added to precipitate gelatin; after removing the supernatant, the gelatin was dried in an oven. Afterward, this gelatin was weighed and redissolved in warm DI water again at a final concentration of 40 mg/mL. To find the optimal parameters for preparing GC, the various amounts/ratios of gelatin solution and cross-linking agent, 25% (v/v) glutaraldehyde (GA), were mixed for further GC preparation. Tested parameters for preparing GC1, GC2, ···, GC7 are listed in Table 1. The mixture was added to a 3 mL syringe following the cryogelation process: frozen at −20 °C for 24 h and then freeze-dried using a lyophilizer (FD4.5–8P-D, Kingmech Scientific, Hsinchu, Taiwan) to obtain GC for further application. For the conductive portion, the gelatin solution and 0.032 mL PEDOT:PSS solution (31 mg/mL) were gently mixed and stirred for 1 min, and then lyophilized as described above. GC with PEDOT:PSS addition was named PGC in this study. Injectability is an important property for cryogel preparation, so it was first tested to evaluate the optimal parameters. The injectability of cryogels was tested: the GC or PGC cylinder was soaked in water to make it fully wetted first. After that, the sample was put into a 3 mL syringe and then pushed out. This test was performed by the same technician, and the integrity and fragmentation were assessed. For the injectability test, if the sample was intact after injection, it was recorded as “Yes.” If not, “No” was scored if it became segmented. For the recovery evaluation, if the sample returned to its original size within 10 s after compression, it was scored as “Yes.”^25^ If not, “No” was recorded for this condition.

**Table 1:** Parameters for Gelatin-Based Cryogel (GC) Synthesis

Sample	Gelatin (mg/mL)	GA (%)	Stirring time (min)	Injectability^a^	Recovery^b^
GC1	15.0	0.50	30	Yes	No
GC2	17.5	0.50	30	Yes	Yes
GC3	20.0	0.50	30	No	No
GC4	17.5	0.25	30	Yes	No
GC5	17.5	1.00	30	No	No
GC6	17.5	0.50	15	No	No
GC7	17.5	0.50	45	Yes	Yes

a Injectability: Yes = Sample remains intact after injection; No = Sample becomes segmented after injection.

b Recovery: Yes = Sample returns to original size within 10 s after compression; No = Sample does not return to original size within 10 s.

### 2.3 Characterization of Cryogels

The optimal parameter for preparing GC was selected as GC2, which was used for further materials and mechanical examination thereafter. The GC2 and PGC2 were fabricated in a cylinder (diameter: 0.88 cm; height: 0.80 cm), and at least three samples per group were used for each examination. The lyophilized GC2 and PGC2 cylinders were weighed on a precision electronic balance (XS-125A, Precisa, Dietikon, Switzerland) as the dry weight (W_1_). Then they were soaked in DI water for water adsorption. The water uptake capacity was calculated by the formula:

$$\mathrm{Water}\;\mathrm{uptake}\;\mathrm{capacity}\left(\%\right)=\frac{\left(W_{2}-W_{1}\right)}{W_{1}}\times 100\%$$

where W_1_ is the dry weight of the samples, and W_2_ is the wet weight of the samples after immersion in DI water for 24 h.^26^

The porosity of the cryogels in ethanol was determined by the formula:

Porosity(%)=(WE2−W1ρethanol)πr2h×100%

where W_1_ is the dry weight of the samples, W_E2_ is the wet weight of the samples after immersion in ethanol for 6 h, r is the inner radius of the sample, and h is the height of the sample.^27^

The chemical/crystal structure of these cryogels (GC2 and PGC2) was analyzed using an X-ray diffractometer (XRD, D2 PHASER, Bruker, Billerica, MA, USA). The 2θ range was measured from 10° to 60° at an increment of 0.02°. All of the samples were dried and attached to a coverslip of a test holder. Chemical bonds of dried cryogels were identified using Fourier-transform infrared spectroscopy (FT-IR, Nicolet iS10, Thermo Fisher Scientific, Waltham, MA, USA) with a wavenumber range of 4000–400 cm^–1^ and a resolution of 4 cm^–1^. The dried cryogels (GC2 and PGC2) were soaked in liquid nitrogen and then fractured to obtain cross sections, and then their microstructure was observed by scanning electron microscopy (SEM, SU3500, Hitachi, Tokyo, Japan) after gold sputter coating, and the pore size of cryogels was measured on SEM images by ImageJ software (1.46r, National Institutes of Health, Bethesda, MD, USA). Then the elemental analysis of PGC (C, N, O, S) was performed under the SEM equipped with the EDAX EDS APPOLLO system. The mechanical properties were determined with a dynamic mechanical analyzer (DMA, Q800, TA Instruments, New Castle, DE, USA). The force ramp rate and upper force limit were respectively set to 10.0 N/min and 18.0 N.

### 2.4 Degradation Evaluation

The degradation of cryogels was tested in PBS with collagenase IV (30 units/mL) addition to mimic the *in vivo* environment. Dry GC2 or PGC2 sample (W1) was placed in a well (24-well plate), then PBS or PBS containing collagenase IV was added into the well separately, and then kept at 37 °C. Samples were then taken out from the well at indicated time points, rinsed with PBS, then dried using a lyophilizer (FD4.5–8P-D, Kingmech Scientific), and finally weighed. The degradation percentage was calculated using the following formula:

$$\mathrm{Degradation}\left(\%\right)=\frac{\left(W_{D2}-W_{1}\right)}{W_{1}}\times 100\%$$

where W_1_ is the initial dry weight of a sample and W_D2_ is the dry weight of a sample after incubation.^26^

### 2.5 Electrochemical Properties Analysis

The electrochemical properties were examined using a potentiostat/galvanostat/impedance analyzer (PalmSens 4, PalmSens, Houten, The Netherlands) with cyclic voltammetry (CV) and electrochemical impedance spectroscopy (EIS) methods in a two-electrode system. Cryogel samples (GC2 and PGC2) were first sufficiently moistened with DI water and then transferred to a well (24-well plate), and two tungsten probes were carefully inserted into each well. The CV parameters were set as follows: t equilibration: 0 s; E begin: 0 s; E vertex1: −1.0 V; E vertex2:1.0 V; E step: 0.01 V; and scan rate: 0.1 V/s. The EIS was conducted with the following parameters: t equilibration: 0 s; E dc: 0.0 V; E ac: 0.01 V; n frequencies: 61/10; max. frequency: 10^6^ Hz; and min frequency: 1.0 Hz.

### 2.6 Drug-Release Examination under Electrical Stimulation

To determine whether the drug release properties were affected by electrical stimulation, GC2 and PGC2 were treated with or without electrical stimulation (ES) for a drug-release test. Cryogels containing Eosin Y (0.3 mg per cryogel) as a model drug were soaked in a PBS solution to evaluate the electrical response and investigate the dye (drug) release behavior of GC2 and PGC2. Cryogel samples were placed in PBS-containing wells (of a 24-well plate), and two activated carbon probes were carefully inserted into each well. Two experiments were performed: In the first experiment, ES (−3 V) and no ES (0 V) conditions were applied to the sample for 15 min per cycle (for a total of 120 min) using the chronoamperometric model controlled by the PalmSens 4 system. In the second experiment, ES was applied to the sample for 15 min, followed by 105 min of incubation without ES, using the chronoamperometric model controlled by the PalmSens 4 system. The following parameters were used: t equilibration: 0 s; E dc: −3.0 V; t interval: 1.0 s; and t run: 900 s. After ES, the supernatant with released dye was collected; then the well was replenished with fresh PBS. The amount of Eosin Y released was quantified by measuring the absorbance at 520 nm on a microplate reader (Epoch 2, BioTek, Winooski, VT, USA). The OD value was compared to the standard curve of Eosin Y for concentration calculations, and the cumulative amount released under multiple ES cycles was also determined.

### 2.7 *In Vitro* Examination

The NIH-3T3 murine embryo fibroblast cell line was obtained from the Bioresource Collection and Research Center (BCRC, Hsinchu, Taiwan). Basal culture medium for maintaining NIH-3T3 cells consisted of DMEM/F-12 supplemented with 10% FBS, 1.2 g/L sodium bicarbonate, and 100 U/mL penicillin/streptomycin.

#### 2.7.1 Cell Viability

The viability of NIH-3T3 cells treated with various samples (GC2 and PGC2) was determined by performing the water-soluble tetrazolium salt (WST)-1 assay. Dry cryogels were soaked in 75% ethanol for 24 h and exposed to UV light for 30 min for sterilization. Samples were rinsed and immersed in FBS-free medium before cell seeding. For the direct contact method, cylindric cryogels were placed in wells of a 96-well plate, and 100 μL of 2 × 10^4^ NIH-3T3 cells/mL was injected into the porous GC via a 23-gauge needle. The same seeding method was also used for the control group (tissue culture). Then, 100 μL of the fresh culture medium was added to the well. Cells seeded in GC were maintained in an incubator at 37 °C with a 5% CO_2_ atmosphere and incubated for 24 h. After that, the culture medium was removed, 220 μL mixed reagent (20 μL Cell Counting Kit (CCK)-8 and 200 μL medium, 1:10 (v/v)) was added to the wells to react with cells in the GC scaffolds, and incubated for 3 h in a 5% CO_2_ incubator at 37 °C.^28^ Afterward, the supernatants were collected and analyzed at a wavelength of 450 nm using a microplate reader (Epoch 2, BioTek, Winooski, VT, USA). For the extraction method, the extraction solution was prepared as follows: One cryogel (GC2 or PGC2) (0.5 mL) was soaked in FBS-free medium (15 mL) and kept at 37 °C for 24 h. NIH-3T3 cells were seeded in a 96-well plate at a density of 5 × 10^3^ cells/well in their culture medium and incubated in a 5% CO2 incubator at 37 °C for 24 h. Then, 100 μL of the extraction solution was added to each well, and cells were further incubated in a 5% CO_2_ incubator at 37 °C for another 24 h. After that, 110 μL of mixed reagent (10 μL CCK-8 and 100 μL medium, 1:10 (v/v)) was added to the wells and incubated at 37 °C for 2 h. Afterward, final solutions were analyzed at a wavelength of 450 nm as previously described.

#### 2.7.2 Live/Dead Staining

After incubating cells with the cryogel extraction solution for 24 h, live/dead reagents (1 μL of a calcein-AM solution for live cell staining and 0.5 μL of a propidium iodide solution for dead cell staining in 1 mL of PBS) were added to each well and incubated for 30 min at 37 °C. Fluorescent images of cells were observed and captured using an inverted microscope (IX71, Olympus, Tokyo, Japan). 70% ethanol was used as the negative control for this test.

### 2.8 Statistical Analysis

All results are presented as the mean ± standard deviation (SD). Each group tested had three to six (*n* = 3–6) samples. A one-way analysis of variance (ANOVA) was performed to compare multiple groups using Excel (Microsoft, Redmond, WA, USA). *p* < 0.05 was considered statistically significant.

## 3 Results

Various parameters for synthesizing injectable PEDOT:PSS-loaded gelatin cryogels (PGC) were first tested, since injectable gels can be easily used for in vivo applications. The process for preparing this cryogel is shown in Figure 1A, and a cross-linking reaction during cryogelation was important. The aldehyde group (−CHO) of GA, the cross-linker, reacted with the amine group (−NH_2_) of gelatin to obtain gelatin peptide chain cross-linked cryogels (GC).

**Figure 1:**
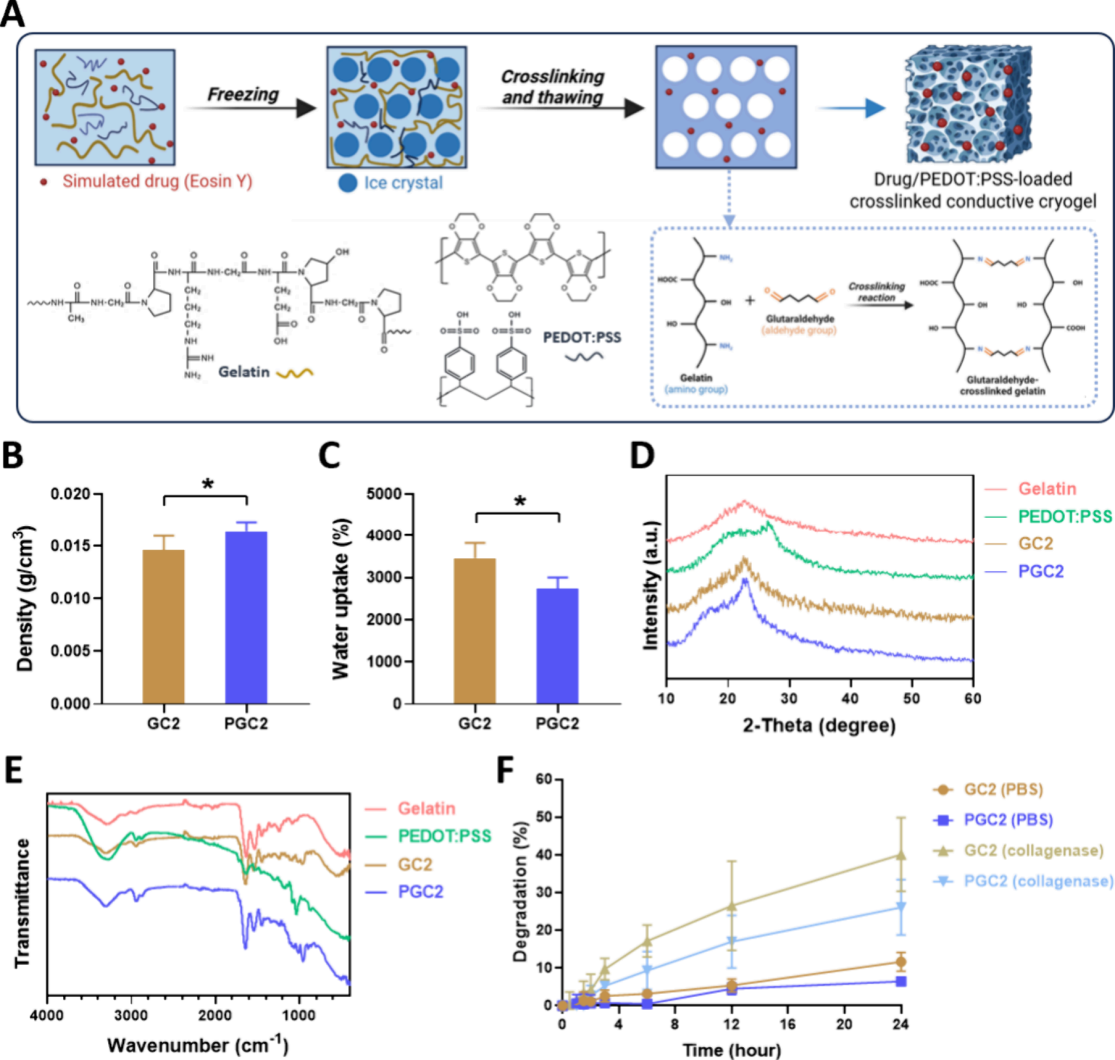
Characterization of gelatin-based cryogels (GC). (A) Schematic drawing of the preparation of GC with cross-linking and PEDOT:PSS addition. (B) Density of GC2 and PGC2 (*n* = 6). (C) Water uptake capacity of cryogels (*n* = 3). (D) XRD patterns of gelatin, PEDOT:PSS, GC2, and PGC2. (E) FTIR spectrum of gelatin, PEDOT:PSS, GC2, and PGC2. (F) Degradation profile of the GC2 and PGC2 in PBS (pH 7.4) with or without collagenase IV addition (*n* = 3). *p < 0.05 for paired comparison.

### 3.1 Characterization of Cryogels for Optimal Parameters Selection

To find the optimal formulation for preparing injectable cryogels, the gelatin amount, GA content, and stirring time for the mixture were evaluated, and the results are shown in Table 1. When the gelatin amount was the variable factor (GC1-GC3), the appearance of GC1 (15.0 mg/mL) could remain intact after passing through the syringe (Injectability: Yes), but it did not expand and return to its original size after injection (Recovery: No); this revealed that GC1 was insufficient to provide the required elasticity for shape recovery after compression. When the amount increased to 20.0 mg/mL (GC3), the injectability of GC3 was not good; some fragments were found after passing through the syringe (Injectability: No), so it was not appropriate. GC2 was prepared at a gelatin concentration of 17.5 mg/mL. Its injectability and recovery tests were both “Yes”, which fulfilled the requirement of a positive result. In addition, when the GA content was the factor affecting GC properties (G2, G4, and G5) for the test, GC4 (0.25% GA) had frayed edges with whisker-like structures, demonstrating that this concentration was insufficient to provide a suitable cross-linking density, leading to structural incompleteness. Therefore, it could not recover within 10 s after compression (Recovery: No). For GC 5 (1.00% GA), higher GA concentration led to excessive cross-linking, resulting in a rigid mechanical structure, causing no injectability (Injectability: No), and it could not recover to its original shape after compression. For the comparison of stirring time (G2, G6, G7), when the stirring time before cryogelation was set to 15 min (GC6), the cryogels exhibited fragmentation after passing through the syringe (Injectability/Recovery: No), whereas both the 30 min (GC2) and 45 min (GC7) gels could pass through the syringe, indicating that a minimum mixing time of 30 min at room temperature was required to achieve uniformity under our experimental conditions. Overall, the GC2 exhibited the most desirable properties for injection and shape recovery, so it was selected as the optimal formulation. The parameters for the suitable cryogel preparation (GC2) were listed as follows: gelatin solution at a final concentration of 17.5 mg/mL, cross-linked with GA (0.50%) at room temperature for 30 min, then processed by cryogelation (−20 °C for 24 h).

After confirming the optimal parameters for GC preparation (GC2), 1 mg/mL PEDOT:PSS was used in the GC2 preparation process to create additional electrical functions for the gel (PGC2). Characterization results of cryogels without (GC2) and with PEDOT:PSS addition (PGC2) are shown in Figures 1B–1F. In Figure 1B, the density of GC2 was 0.0146 ± 0.0013 g/cm^3^. The value shown for PGC2 (with PEDOT:PSS addition) was significantly higher at 0.0165 ± 0.0008 g/cm^3^ (Figure 1B, * *p* < 0.05). In addition, GC2 exhibited an excellent water uptake capacity, with the value reaching 3460% ± 295% (Figure 1C). After PEDOT:PSS addition, the water uptake capacity reduced to 2738% ± 217% (Figure 1C).

XRD results are shown in Figure 1D. Gelatin exhibited a broad peak at 2θ = 20°–22.6°. In the pattern of PEDOT:PSS, a broad peak contributed by PSS was observed at 2θ = 20.8°.^29^ Meanwhile, a more distinct sharp peak at 2θ = 26.6° was found, which was attributed to PEDOT.^30^ After cryogelation, GC2 showed a similar pattern as gelatin, with a broad peak in the range of 20°–22.6°. After PEDOT:PSS addition, a relatively obvious sharp peak of PGC2 appeared at 2θ = 22.6°, differentiating them from GC2.

FTIR results are shown in Figure 1E. Characteristic peaks for gelatin corresponding to O–H and N–H bonds were observed in the 3600–3100 cm^–1^ range, and the characteristic peaks of the C–H bond were observed in the 3000–2800 cm^–1^ range.^31^ Characteristic peaks in the 1650–1550 cm^–1^ range represented C = O and N–H bonds, and characteristic peaks in the 1400–1000 cm^–1^ range represented O–H, C–N, C–C, and C–O bonds.^31^ For PEDOT:PSS, characteristic peaks corresponding to the O–H bond were observed in the 3600–3100 cm^–1^ range, and characteristic peaks of the C–H bond were also observed in the 3000–2800 cm^–1^ range.^32^ The characteristic peaks at 1640 cm^–1^ represented the C = C bond, the characteristic peak in the 1300–1000 cm^–1^ range was contributed by O–H, S = O, C–C, and C–O bonds, and the peak in the 900–700 cm^–1^ range represented S–O, C–H, and C–S bonds.^30,32^ In the results of GC2 and PGC2, no new distinct peaks were observed due to the overlap of signals from gelatin and PEDOT:PSS. Nevertheless, the absence of peak loss suggests the structural integrity was maintained during synthesis.

Cryogel degradation profiles are shown in Figure 1F. In the PBS environment, GC2 showed a slow degradation rate; after 24 h, the GC2 degradation percentage was 11.7% ± 2.0%. The PGC2 group (with added PEDOT:PSS) revealed a slightly slower degradation rate of 6.4% ± 0.5% after 24 h. When treated with collagenase IV (mimicking an *in vivo* enzymatic digestion), the degradation rates for both GC2 and PGC2 increased, with degradation rates of GC2 and PGC2, respectively, increasing to 40.2% ± 7.9% and 26.1% ± 6.0% after 24 h. The degradation tendencies in both conditions were similar: GC2 > PGC2.

### 3.2 Macro- and Microstructure of Cryogels

For macro-/microstructural observations, photos acquired by a digital camera and SEM imaging are provided in Figures 2A–2B. The appearances of GC2 and PGC2 at different stages of preparation are shown in Figure 2A. GC2 had a slight yellow color in both the cryogelation and lyophilization processes. With the addition of PEDOT:PSS, the color of cryogels turned blue (PGC2). Both GC2 and PGC2 were formed in a syringe with cylindrical shapes, and their shapes were still maintained as cylinders after absorbing water (hydration). The microstructures of GC2 and PGC2 observed in SEM images revealed that both GC2 and PGC2 exhibited porous structures with lamellar walls and interconnected pores (Figure 2B). For macro-/microstructural analysis, a gelatin hydrogel (GH) prepared without cryogelation was also examined by optical imaging and SEM for comparison (see Supporting Information). Nonsolidification and nanoscale pore structures were observed in the GH and PGH (PEDOT:PSS addition) (Figures S1A–S1B). The EDS elemental mapping of PGC2 is shown in Figure 2C, and its EDS spectrum is provided in Figure S2. The presence of C, N, O, and S confirmed the integration of PEDOT:PSS with GC2, where the N signal originated from gelatin and the S signal from PEDOT:PSS. The major pore size distributions of GC2 (Figure 2D) and PGC2 (Figure 2E) were in the range of 90–190 μm, with no obvious difference between GC2 and PGC2. The porosity of the cryogel, shown in percentage, is given in Figure 2F: GC2 exhibited a porosity of 51.8% ± 2.6%. With the addition of PEDOT:PSS, the porosity of PGC2 increased to 72.4% ± 2.9% (*p < 0.05).

**Figure 2:**
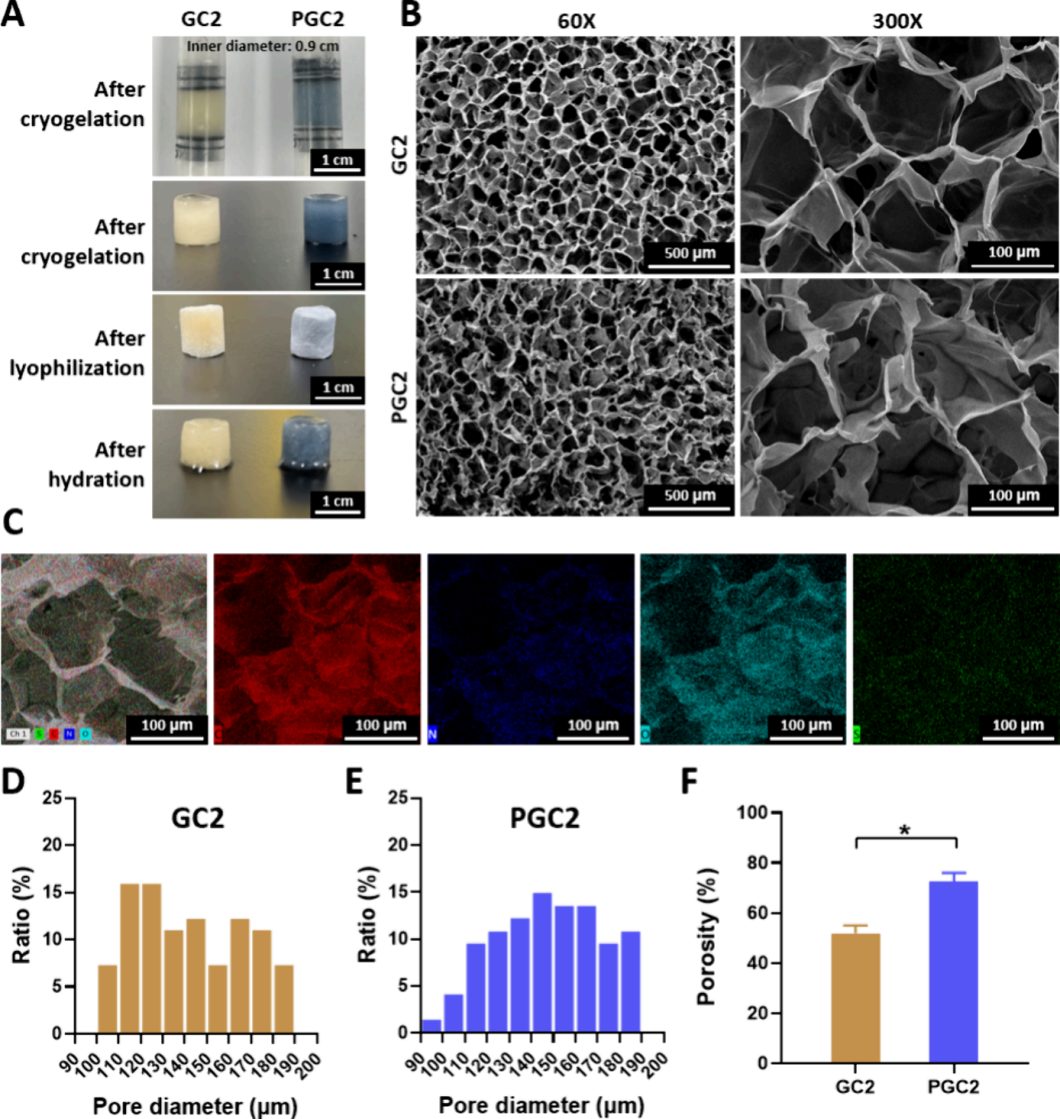
Appearance and microstructure of gelatin-based cryogels. (A) Photos of cryogels after cryogelation in a syringe, lyophilization, and hydration. Cryogels were prepared using 3 mL syringes with an inner diameter of 0.9 cm. (B) SEM images of GC2 and PGC2 under 60X and 300× magnification. (C) Images of EDS elemental mapping of PGC2 (C, N, O, and S). Pore diameter distribution of (D) GC2 and (E) PGC2. (F) Porosity of the GC2 and PGC2 (*n* = 3). *p < 0.05 for paired comparison.

### 3.3 Elastic-Like Property of Cryogels

For further application in biological environments as an injectable material, compression tests of the cryogels were performed in water-containing conditions. Results of cryogel compression tests are shown in Figure 3A, which include photographs of applying forces vertically and parallel to the cylinder. It was observed that both GC2 and PGC2 could be squeezed, and pore-containing water could be pushed out (compressed state). When the force was released, these two cryogels quickly recovered to their original shape (recovery state). In the photos in Figure 3B, results of the injectability tests are given, where an injection was performed through a 3 mL syringe. When PGC2 was pushed out of the syringe, it was squeezed into a very small size to pass through the fluid path of the syringe with a narrow diameter (arrow indicated). Additionally, it was able to quickly return to its original size within a few seconds (recovery period/state). No fragmentation or debris of the cryogels was found after injection, which is one advantage of cryogels. A similar condition was also observed in the GC2 test. The same method was also performed on gelatin hydrogel (GH) made of the same composition but without cryogelation (see Supporting Information S1. Materials and Methods). When injecting the GH and PEDOT:PSS-loaded GH (PGH) out of the syringe, it was like a mud dispersion in the water, both observed in GH and PGH groups (Figure S3).

**Figure 3:**
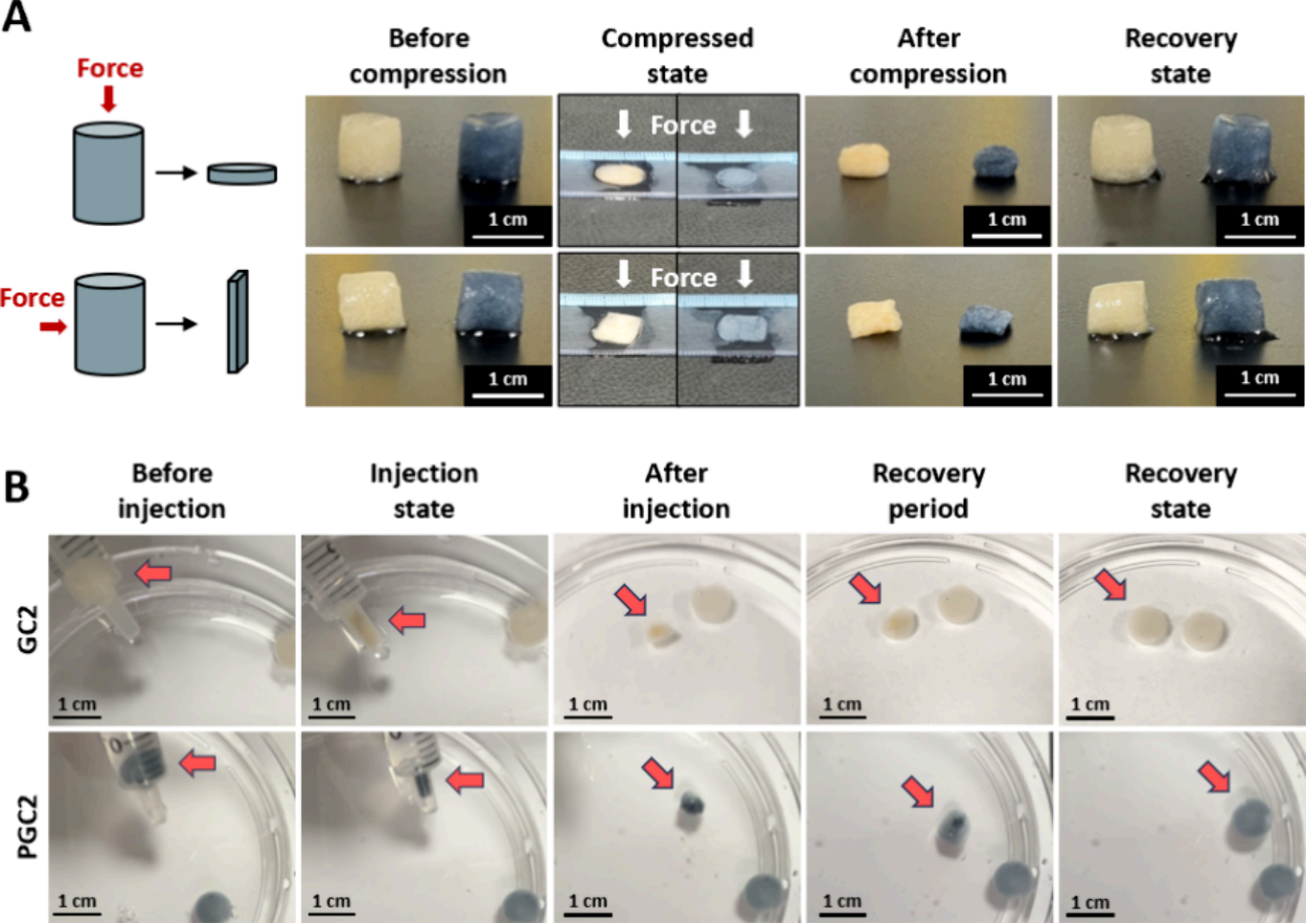
Compressibility and injectability of the cryogels. (A) Photographs of the compression test with size/shape changes in GC2 and PGC2. (B) Sequential photographs revealed the injection process and changes in GC2 and PGC2. Injection state: sample was pushed to pass through the neck of the syringe. Recovery state: sample returned to its original shape and size.

### 3.4 Mechanical Properties of the Cryogels

A dynamic mechanical analyzer (DMA) was used to measure the mechanical properties. Figures 4A–4B show the force loading on the vertical and parallel sides of the GC2 and PGC2. Results are shown in Figures 4C–4D. Under the same loading force, whether compressed from the vertical (Figure 4C) or parallel directions (Figure 4D), PGC2 exhibited a higher strain value than GC2 in both directions. For example, the PGC2 in Figure 4D (48% strain at 0.2 MPa of stress) was higher than that of GC2 (34% strain at 0.2 MPa of stress), indicating that PGC2 had a lower compressive modulus, meaning it was softer and easier to deform. Repeated cyclic tests at a 50% strain (vertical sides) are shown in Figures 4E–4F. GC2 exhibited similar curves across the 10th, 20th, and 30th cycles (Figure 4E), and PGC2 also showed consistency across these cycles (Figure 4F), suggesting the good elasticity of GC2 and PGC2.

**Figure 4:**
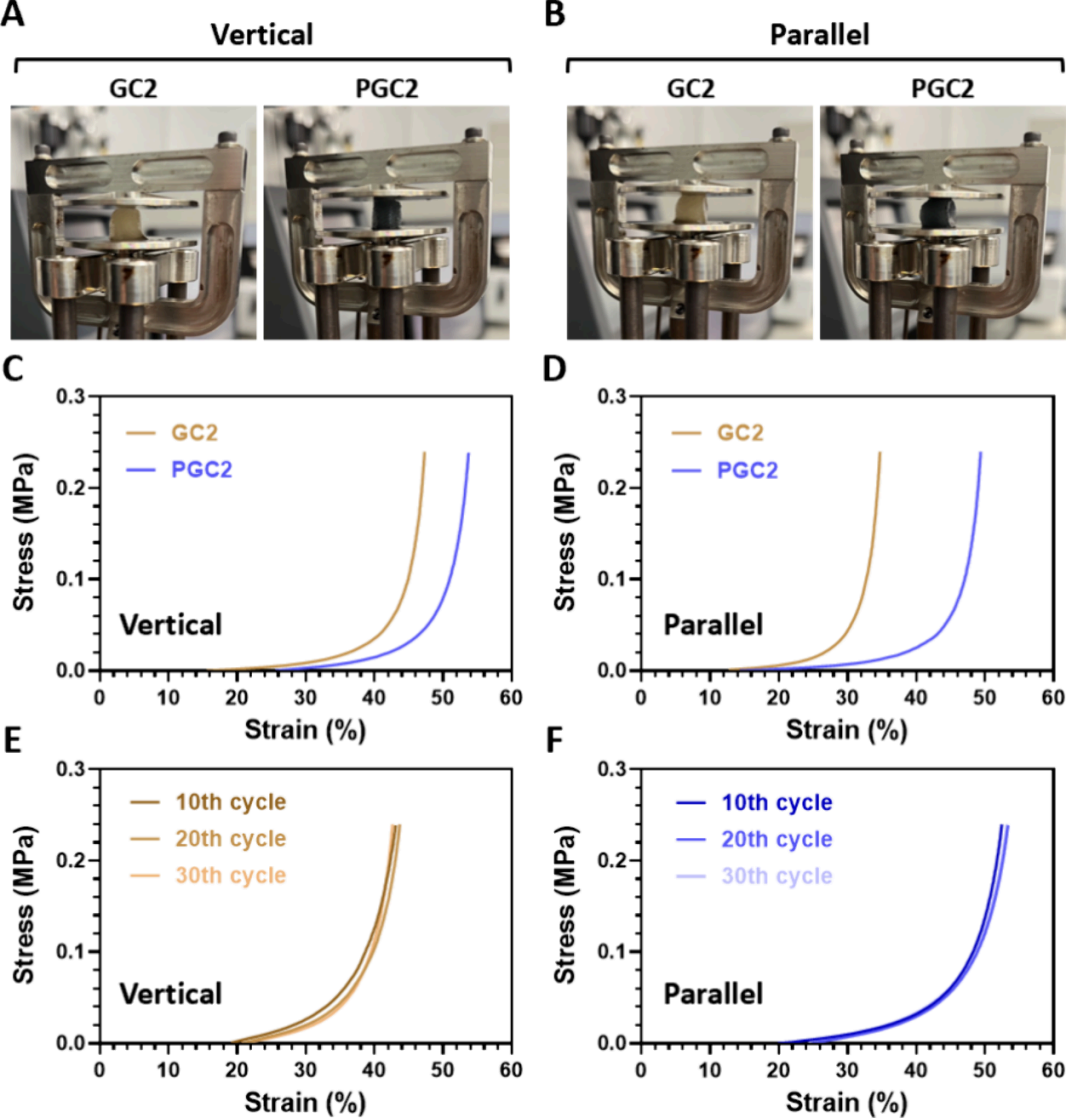
Mechanical properties of the cryogels. Photos showing the cryogels examined under (A) vertical-side compression and (B) parallel-side compression. The stress–strain curves of GC2 and PGC2 under (C) vertical-side compression and (D) parallel-side compression. The cyclic compressive stress–strain curves of (E) GC2 and (F) PGC2 after different cycles under vertical-side compression.

### 3.5 Conductive Properties Endowed by PEDOT:PSS in PGC2 for Drug Release

The electrical property was examined by the CV method, and the results are shown in Figure 5A. When scanned at the potential range of – 1.0 to 1.0 V, the gelatin solution (17.5 mg/mL) demonstrated a typical CV curve, with an oxidation current of 2.95 ± 0.02 μA and a reduction current of −3.07 ± 0.04 μA; this can be attributed to electroactive amino acids, such as tyrosine and tryptophan, which exhibit redox properties and can undergo redox reactions at particular potentials.^33^ After cryogelation, GC2 demonstrated a similar result as gelatin, with a slightly increased oxidation current of 3.48 ± 0.01 μA and a reduction current of −3.39 ± 0.01 μA (Figure 5A). The changes in oxidation/reduction current may be due to the formation of a 3D structure that alters electron transport pathways, thereby affecting redox behavior. The pattern of PEDOT:PSS (1 mg/mL) revealed a typical CV curve with an oxidation current of 2.07 ± 0.01 μA and a reduction current of −2.09 ± 0.01 μA. The PGC2 demonstrated a higher oxidation current of 3.93 ± 0.01 μA and a reduction current of −3.85 ± 0.01 μA (Figure 5A).

**Figure 5:**
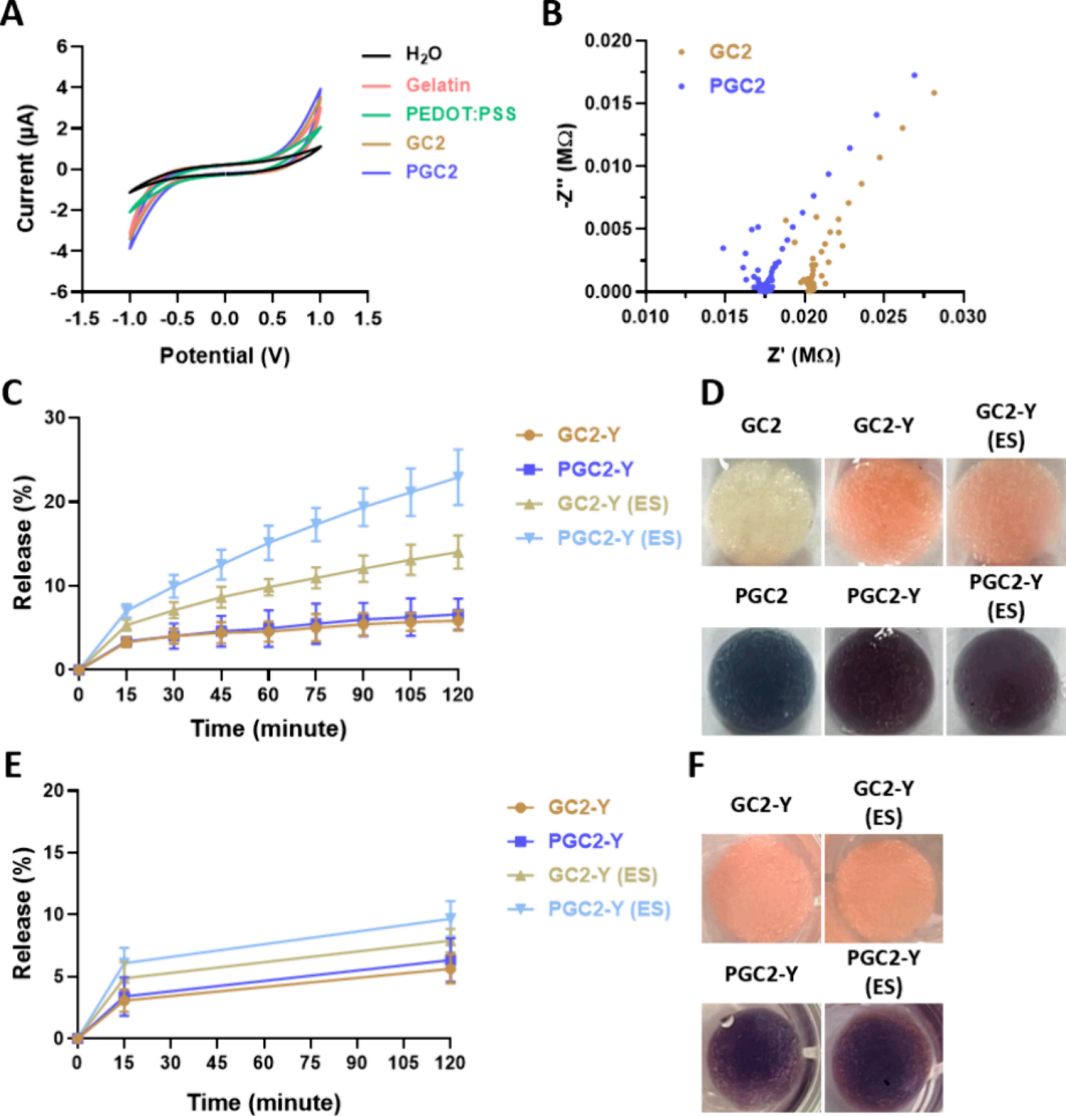
Electrochemical properties and dye release properties of the cryogels. (A) Cyclic voltammograms of different samples were recorded from – 1.0 to 1.0 V at a scan rate of 0.1 V/s in DI water. (B) Nyquist plot of the GC2 and PGC2 in DI water. (C) The release profile of Eosin Y from GC2-Y and PGC2-Y after 8 cycles of ES (15 min per cycle) with or without ES in PBS. (D) Photographs of the GC2-Y and PGC2-Y after 8 cycles of ES. (E) The release profile of Eosin Y from GC2-Y and PGC2-Y after applying ES for 15 min, followed by incubation for an additional 105 min in PBS without ES. (F) Photographs of the GC2-Y and PGC2-Y after ES and incubation.

The results of impedance spectroscopy (EIS) are shown in Figure 5B. GC2 demonstrated a certain level of conductivity with a real resistance of 0.0208 ± 0.0007 MΩ (n = 3). The conductivity of GC2 may be attributed to the presence of electroactive amino acids and the formed 3D network structure that facilitates charge transport.^33,34^ After incorporating PEDOT:PSS, a decrease in real resistance was observed, with a value of 0.0174 ± 0.0001 MΩ (n = 3), confirming the effective enhancement of conductivity by adding PEDOT:PSS. The PGC2 possessed conductive properties that could respond to electrical signals.

Using electrical stimulation as a control factor for drug-delivery applications needed to be demonstrated. Results of the drug-release test performed under electrical stimulation (ES) are shown in Figures 5C-5F. In Figure 5C, release of Eosin Y from GC2 (GC2-Y) in the first 15 min was observed in all groups (without ES), which was attributed to the small amount of dye adsorbed onto the cryogel surfaces. For up to 2 h, Eosin Y release rates in the GC2-Y and PGC2-Y groups were all low (<5%). The release rate of GC2-Y significantly increased when applying electrical stimulation (ES), and a final cumulative release rate of 14.0% ± 1.6% after 120 min of treatment (with eight cycles of ES) was recorded. When PEDOT:PSS was added to GC2-Y (PGC2-Y) and ES was applied, the release curve of PGC2-Y demonstrated a faster release profile, with a higher initial release of 7.0%, ultimately reaching 22.9% ± 2.7% after 120 min of treatment (Figure 5C, PGC2-Y, ES). The slope of PGC2-Y was 0.151 %/min between 15 and 120 min. Color changes from pink (GC2-Y) to light pink (GC2-Y, ES) due to Eosin Y being released from GC2 were observed (Figure 5D). Although the dark blue of PGC2-Y hindered the observation of dye release, color and tone changes in the PGC2-Y were also found after ES. In Figure 5E, the first 15 min of the release test was performed under ES conditions, then ES was removed for a diffusion release test. The release profiles of GC2-Y and PGC2-Y resembled those in Figure 5C after 15 min, and once the ES was removed, the subsequent release rates slowed down, with slopes similar to those of the unstimulated groups. The slope of PGC2-Y was 0.034 %/min between 15 and 120 min. This suggests that the system exhibits a turn-on/turn-off release behavior in response to electrical stimulation for drug release control.

### 3.6 Good Biocompatibility of PEDOT:PSS-Containing Cryogels

To evaluate the cytotoxicity of these cryogels, two methods (direct contact and extraction) were adopted via NIH-3T3 cell cultivation, and the WST-1 assay and Live/Dead staining assay were performed. Results of the direct contact method (cells seeded into cryogels) are shown in Figure 6A. For the control group, cells were also seeded into the culture well by a 23-gauge needle (the same as the cryogel scaffold treatment) to diminish the doubt that cells were damaged when passing through the tiny needle. The cell viability was 100.6% (n = 4); this value did not differ from cells seeded in the well using a 200-μL tip (100.0%), which confirmed that the injection process did not affect cell viability. The cell viability value of GC2 was higher (123.9% ± 6.0%); this may be attributed to the gelatin nature and 3D porous structure of GC2, providing a biomimetic environment as an ECM, facilitating cell attachment and proliferation, which was superior to a 2D culture dish.

**Figure 6:**
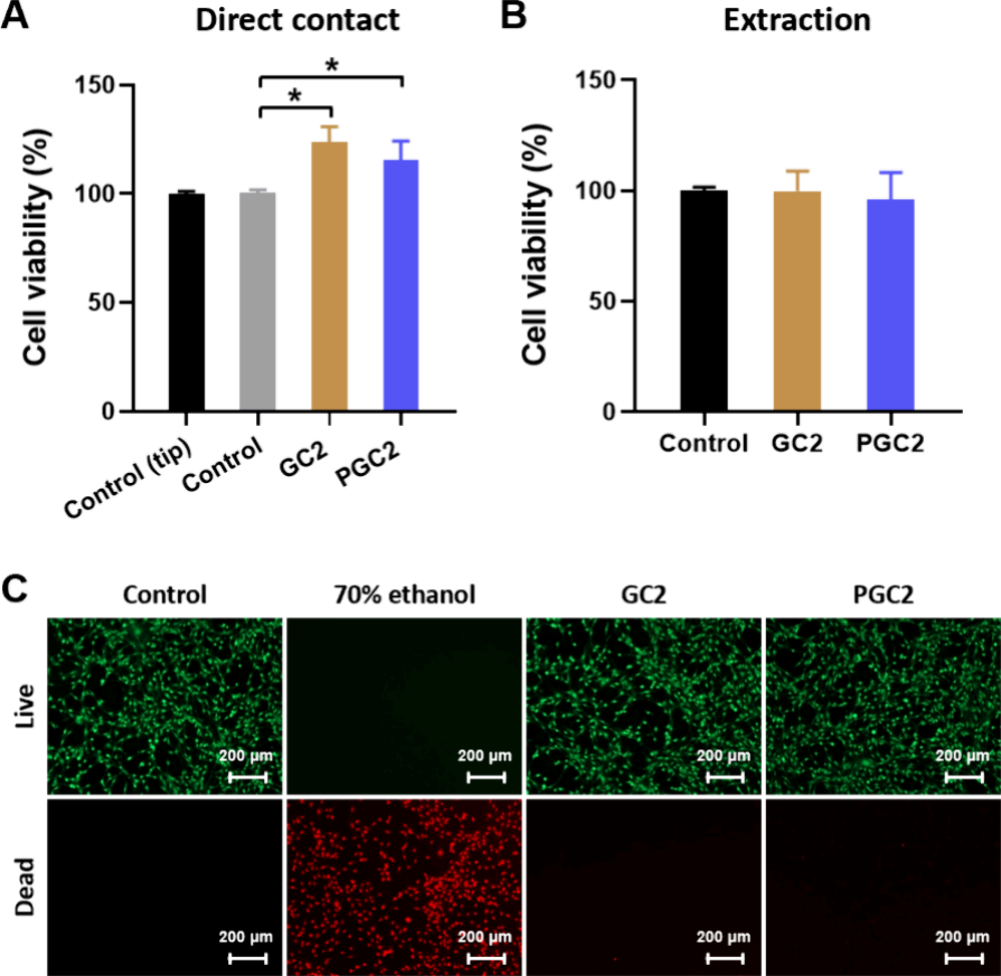
*In vitro* cytocompatibility. (A) The cell viability of NIH-3T3 cultured in cryogels for 24 h (direct contact mode, *n* = 4). (B) The cell viability of NIH-3T3 incubated with the cryogel extraction medium for 24 h (extraction mode, *n* = 5). (C) Live/Dead staining of NIH-3T3 incubated with control (culture medium), 70% ethanol, and the extraction medium of GC2 and PGC2 for 24 h. Scale bar = 200 μm. *p < 0.05 for paired comparison.

After adding PEDOT:PSS, the PGC2-treated cells revealed the cell viability at 115.5% ± 7.5%, which was still better than the culture well but similar to the GC2 group. Results of the extraction test are shown in Figures 6B–6C. The cell viability of NIH-3T3 cells of the GC2-treated group showed good cytocompatibility (99.8% ± 8.0%), with no significant difference compared to the control group (100.0% ± 1.3%). After adding PEDOT:PSS, the PGC2 group also showed good cell viability (96.2% ± 10.7%). According to the cytocompatibility standards of ISO 10993–5, these results indicated good cytocompatibility of GC2 and PGC2 (>70%).^35^ Similar results were obtained in the Live/Dead staining assay. As shown in Figure 6C, huge amounts of dead cells (red fluorescence) were observed in the 70% ethanol group (negative control), whereas mainly viable cells (green fluorescence) were observed in the control, GC2, and PGC2 groups.

## 4 Discussion

In the field of tissue engineering, scaffolds play an important role in cell growth and tissue reconstruction. Scaffolds with a suitable composition and proper pore size/porosity, degradability, mechanical strength, and biocompatibility are design factors to fabricate suitable scaffolds with appropriate tissue responses. Hydrogels with those above-mentioned advantages can be suitable scaffolds for application in tissue engineering. However, the low mechanical strength and low elasticity of hydrogels limit their application. Recently, cryogels were studied due to their higher mechanical strength, elasticity, and structural flexibility.^5^ Moreover, their interconnected microporous structure, formed by ice crystals during the cryogelation process, provides a favorable microenvironment in which cells can reside, which promotes cell adhesion, growth, migration, and differentiation. Cryogels can be a good scaffold applied in regenerative medicine.

The raw material chosen to prepare cryogels in this study was gelatin, the denatured form of collagen, the major protein in the ECM. Gelatin has biocompatibility, biodegradability, and low antigenicity.^36^ The peptide chain of gelatin possesses arginine-glycine-aspartic acid (RGD) sequences, which facilitate cell adhesion through integrin binding.^36,37^ In addition, its susceptibility to enzymatic degradation by matrix metalloproteinases (MMPs) allows for controlled biodegradation and tissue remodeling.^36,37^ Studies have shown that the cross-linking degree affects the morphology, porosity, water uptake capacity, and mechanical properties of gels.^38^ Higher cross-linking agent concentrations may lead to excessive cross-linking, resulting in a rigid mechanical structure that fractures upon compression.^39^ In contrast, a lower degree of cross-linking could result in insufficient mechanical properties, leading to an incomplete structure and failure to recover its original shape after compression.^39^ In this study, we investigated factors influencing the cross-linking reaction (gelatin/GA concentrations and reaction times) and evaluated optimal parameters for GC preparation based on injectability and compressibility when shape recovers to its original form. A narrow concentration range suitable for synthesizing gelatin-based cryogels (GC) with desirable mechanical properties was observed, with gelatin and GA concentrations set at 17.5 mg/mL and 0.5% for preparing the GC, as shown in GC2 in Table 1.

To make cryogels as multifunctional materials for application in some specific tissues, such as neurons, muscles, biosensing, or drug delivery systems, conductive polymers were added to the gels. Among various conductive polymers, PPy, PANI, and PEDOT:PSS generally exhibit relatively good conductivity. For instance, poly­(4-vinylpyridine) (p­(4-VP)) cryogels were synthesized and incorporated with polyaniline (p­(An)), PPy, and polythiophene (p­(Th)) via *in situ* oxidative polymerization. That cryogel, p­(4-VP)/p­(Th), exhibited high conductivity and was applied for gas sensing and dye movement.^40^ In another study, polysaccharide-based cryogels were developed using carrageenan and sodium alginate with PPy incorporation. The PPy-cryogel-modified electrodes demonstrated enhanced electron transfer and also contributed to DNA immobilization, making them suitable for use as electrochemical biosensors for DNA detection.^41^ A gelatin-based cryogel with a conductive poly­(ethylene dioxythiophene)/polystyrenesulfonate matrix was developed and combined with Ca/Mn codoped barium titanate (CMBT) nanofibers as the piezoelectric filler.^42^ This conductive scaffold (Gel-PD-CMBT) demonstrated a greater capacity to promote cellular osteogenic differentiation *in vitro* and neo-bone formation *in vivo* by restoring the local electroactive microenvironment, offering an ideal platform for electrophysiological bone regeneration.^42^ These findings demonstrate the advantages of combining cryogels with conductive polymers!

As to the characterization of cryogel properties in this study, the density of PGC2 increased with the incorporation of PEDOT:PSS (Figure 1B), which is reasonable because PEDOT:PSS was added at a fixed volume. A porous scaffold provides the matrix that supports cell attachment and growth and also helps with liquid flow for nutrient supply, metabolic waste movement, vascularization, and supporting tissue formation and remodeling.^43^ It was reported that the optimal pore size of a scaffold applied for chondrocyte growth and cartilage regeneration is in the range of 50–250 μm.^10,44^ Cryogels prepared in this study were approximately 90–190 μm (Figures 2D–2E). Therefore, GC2 and PGC2 may also be applied for cartilage regeneration in the future. For applications in biological systems, the water content of the scaffold plays a critical role and is influenced by its porosity.^44^ The porosity and pore size are critical criteria for tissue engineering scaffolds; they not only affect nutrient and metabolic waste exchange in scaffolds but also influence the degradation rate.^45^ Scaffolds with a higher water-uptake capacity have greater hydrophilicity, which is more conducive to cell attachment and proliferation.^46^ Both GC2 and PGC2 demonstrated exceptional water uptake capacities (Figure 2C), indicating that GC2 and PGC2 could effectively maintain a wettable microenvironment, supporting the transport of essential biomolecules and waste products in culture medium or body fluid.

The addition of PEDOT:PSS to the polymeric network as a spatial hindrance resulted in a lower degree of cross-linking; therefore, a loose chain structure was revealed as the porosity increased (Figure 2F). Cryogels with a low cross-linking degree have insufficient strength to resist the expansion of ice crystals during the cryogelation process.^39^ Due to the lower cross-linking degree of PGC2, a lower compressive modulus was observed when compared to GC2; PGC2 was softer (Figures 4C–4D). XRD results (Figure 1D) revealed that the PGC2 pattern showed a sharper peak at 2θ = 22.6°, suggesting a change in the polymer structural arrangement due to PEDOT:PSS incorporation. The successful incorporation of PEDOT:PSS was observed by a color change (light yellow to blue, Figure 2A) and the S signal from the EDS spectrum (Figure S2).

Biodegradable scaffolds have wide applications in the biomedical field due to their tunability, which allows them to be tailored to different application needs.^47^ Scaffolds with higher porosity and faster degradation rates are more suitable for cartilage regeneration.^48^ When the degradation rate aligns with the formation of new tissue, biodegradable scaffolds further support tissue regeneration.^49^ Degradation rates of both GC2 and PGC2 increased when digested by collagenase IV (Figure 1F), indicating that these two cryogels are biodegradable via enzyme digestion, making them suitable for use as *in vivo* implants.

Minimally invasive surgery involves small incisions, which help reduce the risk of postoperative infections, alleviate pain, and promote faster recovery.^50,51^ Injectable hydrogels and cryogels can help achieve those demands.^51,52^ Cryogels, which are flexible and can be compressed without causing significant mechanical damage, can potentially be applied in minimally invasive surgeries. Shape recovery in cryogels is mainly due to their elastic polymer network and interconnected macroporous structure.^53^ Under compression, water is squeezed out to relieve stress as the network deforms. Once the stress is released, the stored elastic energy drives the cryogel to recover its shape.^53^ GC2 and PGC2 underwent deformation during compression. However, regardless of whether they were compressed from the top (Figure 3A, vertical), the sides (Figure 3A, parallel), or through a syringe (Figure 3B), these two cryogels were squeezed and returned to their original shape and size after rehydration—most importantly, without any obvious damage. These results indicate that the GC2 and PGC2 synthesized herein could effectively remain *in situ* after injection into a targeted implanted area, and no fragments were found after exclusion. Moreover, the shape recovery ability suggests these cryogels could extend and occupy the damaged regions, which may be a good point for applying them in cartilage defect repair or hemostasis.^54^

Electrochemical properties are a crucial aspect of this study. Incorporating a conductive polymer into a scaffold to enhance its conductivity not only allows the scaffold to be used as an electrically responsive element but also presents the potential to serve as a medium for electrical stimulation therapy, thereby helping reduce inflammation, facilitate wound healing, and improve cell function.^17,55^ From the CV results (Figure 5A), we can confirm that PEDOT:PSS indeed possessed electron-transfer capabilities, and gelatin also exhibited low electron-transfer capabilities due to its electroactive properties from the side chain of its peptide sequence. EIS results further confirmed that GC2 had a certain level of conductivity, which was further enhanced by the addition of PEDOT:PSS (Figure 5B). These findings are supported by the results of the drug release profile (Figures 5C–5D). For the drug release test, Eosin Y, a common dye, was selected for drug release simulation since it is easy to observe/evaluate dye release. The Eosin loaded GC2 (GC2-Y) showed a significantly higher release of Eosin Y when using electrical stimulation (ES) at −3 V compared to that without ES treatment. The release pattern of PGC2-Y demonstrated a greater amount of Eosin Y release compared to GC2-Y (no PEDOT:PSS). The enhanced release of Eosin Y under ES can be attributed to the electrostatic repulsion between carboxylate anions (−COO^–^) from both Eosin Y and gelatin. This repulsion was triggered by two phenomena (Figure S4A): the dissociation of carboxyl groups (−COOH) into carboxylate anions (−COO^–^) under a negative potential and the electrolysis of water, which generates hydroxyl ions (OH^–^) that create a basic environment, further promoting the deprotonation of carboxyl groups.^20^ In addition, SEM images revealed that the microstructure of PGC2 was changed after electrical stimulation: the pores became more irregular, and the pore structure, ice-crystal-like space, was collapsed (Figure S5). This should be resulted from deformation of negatively charged gelatin in response to electrostatic repulsion, particularly in the more flexible and conductive PGC2. Furthermore, a single-ES experiment (Figure 5E) demonstrated the turn-on/turn-off behavior. These findings indicate that PGC2 formed an electrically responsive scaffold that could be employed for controlled drug-release applications.

Finally, since the GC2 and PGC2 are intended for use as biomedical scaffolds, we conducted *in vitro* cytocompatibility experiments to ensure their safety before going for in vivo tests. Whether through direct cell culture on these cryogels or indirect coculture with their extract solutions, both approaches were nontoxic (Figures 6A–6C), and we found that NIH-3T3 cells cultured on GC2 or PGC2 exhibited higher cell viability than those cultured on TCPS in a 2D environment. These results are consistent with other literature, mainly because the amino acid composition of gelatin can mimic the ECM, and the 3D porous structure of the cryogels provides favorable conditions for cellular behavior.^27^ PEDOT:PSS also possesses biocompatibility,^56^ and the incorporation of PEDOT:PSS into gelatin-based hydrogels has been shown to enhance astrocyte adhesion and long-term viability, together creating an improved biomimetic environment,^57^ indicating that such materials hold great potential for biomedical applications.

## 5 Conclusions

In this study, injectable PEDOT:PSS-loaded gelatin cryogels (PGC2) were successfully prepared and served as conductive scaffolds for tissue regeneration which also can respond to electrical stimulation for controlled drug release. Characterization results demonstrated that PGC2 possessed a broad range of water uptake, reversible squeezability, degradability, and high porosity, forming numerous interconnected pores with suitable pore sizes for cell growth. Moreover, with a negative potential input, conductive PGC2 demonstrated no toxicity to cells and also helped drug release when ions on the polymer chain responded to electrical stimulation. Overall, with the addition of PEDOT:PSS to the cryogels, the conductive cryogels (PGC2) have the potential to be an ideal scaffold that can be shaped and injected via a syringe for application in tissue regeneration such as neuron, bone, cartilage, and muscle. It is also a controlled drug-delivery system responsive to electrical stimulation.

## Supplementary Material



## Notes

Additional methods (preparation of PEDOT:PSS-loaded gelatin hydrogels (PGH) and characterization of hydrogels) and results (appearance and microstructure of the hydrogels, EDS spectrum of PGC2, injectability of the hydrogel, schematic illustration of the electrically responsive release mechanism, full-wavelength scan/standard curve of Eosin Y, and SEM images of GC2 and PGC2 before and after electrical stimulation) (PDF)
