# Supplementary material for: PEDOT:PSS-Loaded Gelatin Cryogels for Electrically Controlled Drug Release
Source: ACS Omega. 2025 Jul 17;10(29):31693–705. doi: 10.1021/acsomega.5c02628 (PMC12311670; doi:10.1021/acsomega.5c02628)
Supplement: Supplementary file 1 [file ao5c02628_si_001.pdf]

Supporting Information

PEDOT:PSS-Loaded Gelatin Cryogels for Electrically Controlled Drug Release

Huai-An Chen[1,†], Ching-Yu Lee[2,3,†], Bo-Jun Huang[1], Kai-Yun Tu[4], Pei-Hsuan Sung[4], Le Ngoc Hoang[1], Ching-Li Tseng[1,5,6,7]*

[1]Graduate Institute of Biomedical Materials and Tissue Engineering, College of Biomedical Engineering, Taipei Medical University, Shuang-Ho Campus, No.301, Yuantong Road, Zhonghe District, New Taipei City 23564, Taiwan.

[2]Department of Orthopedics, Taipei Medical University Hospital, No. 252, Wuxing Street, Xinyi District, Taipei City 11031, Taiwan.

[3]Department of Orthopedics, School of Medicine, College of Medicine, Taipei Medical University, No. 250, Wuxing Street, Xinyi District, Taipei City 11031, Taiwan.

[4]Taipei Municipal Zhongshan Girls High School, No. 141, Section 2, Changan East Road, Zhongshan District, Taipei City 10455, Taiwan.

[5]International Ph.D. Program in Biomedical Engineering, College of Biomedical Engineering, Taipei Medical University, Shuang-Ho Campus, No.301, Yuantong Road, Zhonghe District, New Taipei City 23564, Taiwan.

[6]Research Center of Biomedical Devices, College of Biomedical Engineering, Taipei Medical University, No. 250, Wuxing Street, Xinyi District, Taipei City 11031, Taiwan.

[7]International Ph.D. Program in Cell Therapy and Regenerative Medicine, College of Medicine, Taipei Medical University, No. 250, Wuxing Street, Xinyi District, Taipei City 11031, Taiwan.

[†] These authors contributed equally to this work.

*Correspondence:

Ching-Li Tseng, Tel: 886-2-66202589 ext.15618, Email: chingli@tmu.edu.tw

S1. Material and Methods

S1.1 Preparation of PEDOT:PSS-loaded gelatin hydrogels (PGH)

Hydrogels were prepared by gelation. First, gelatin underwent a desolvation method for purification by dissolving it in deionized (DI) water, heating to 40°C for dissolution, adding excess acetone, removing the supernatant, and drying in an oven. Afterward, the gelatin was weighed and redissolved in warm DI water at a final concentration of 40 mg/mL. Next, a cross-linking agent, 20 μL 25% (v/v) glutaraldehyde (GA), and 0.543 mL DI water were added to 0.437 mL of gelatin solution and stirred at 300 rpm and room temperature for 30 min. The mixture was added to a 3-mL syringe and incubated at 4°C for 24 h to obtain gelatin hydrogels (GH). For the conductive portion, the gelatin solution and 0.032 mL PEDOT:PSS solution (31 mg/mL, pre-sonicated for 2 min) were gently mixed and stirred for 1 min, and then proceeded for gelation as previously described. GH with added PEDOT:PSS were named PGH in this study.

S1.2 Characterization of hydrogels

The microstructure and morphology of GH and PGH were determined by scanning electron microscopy (SEM, SU3500, Hitachi, Tokyo, Japan). After gelation, GH and PGH were frozen in liquid nitrogen for 5 min, and then freeze-dried by using a lyophilizer (FD4.5-8P-D, Kingmech Scientific Co., Ltd., Taiwan). Before analysis, the dried GH and PGH were frozen in liquid nitrogen and then fractured to obtain the cross-section. The injectability of hydrogels was tested: the GH and PGH cylinders were transferred into a 3-mL syringe and pushed out. This test was performed by the same technician.

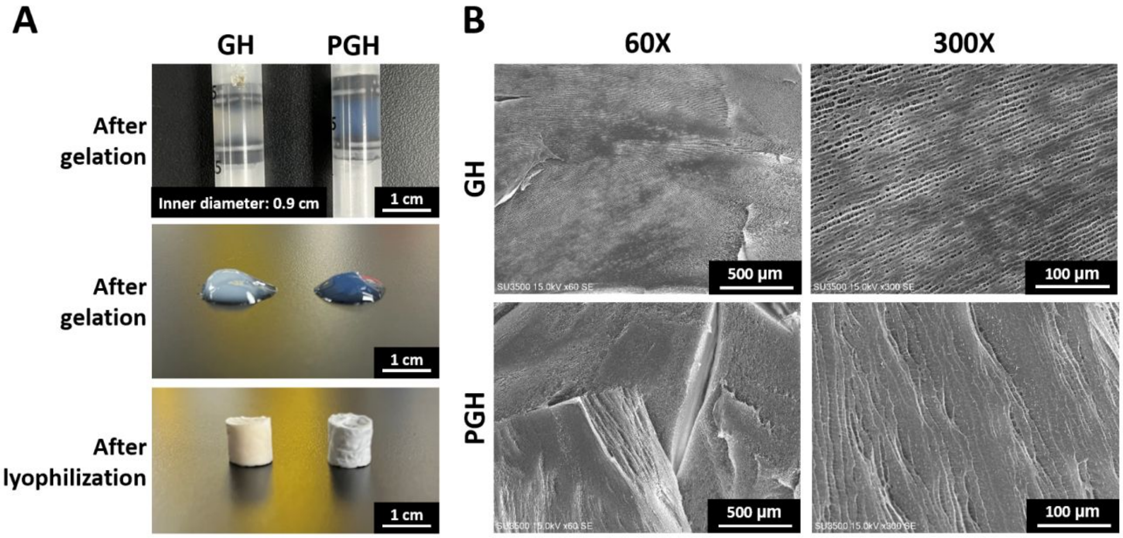

Figure S1. Appearance and microstructure of the hydrogels. (A) Optical images of gelatin-based hydrogels (GH) and PEDOT:PSS-loaded gelatin-based hydrogels (PGH) after gelation and lyophilization. Hydrogels were prepared using 3 mL syringes with an inner diameter of approximately 9 mm and an outer diameter of approximately 11 mm. (B) SEM images of GH and PGH under different magnification.

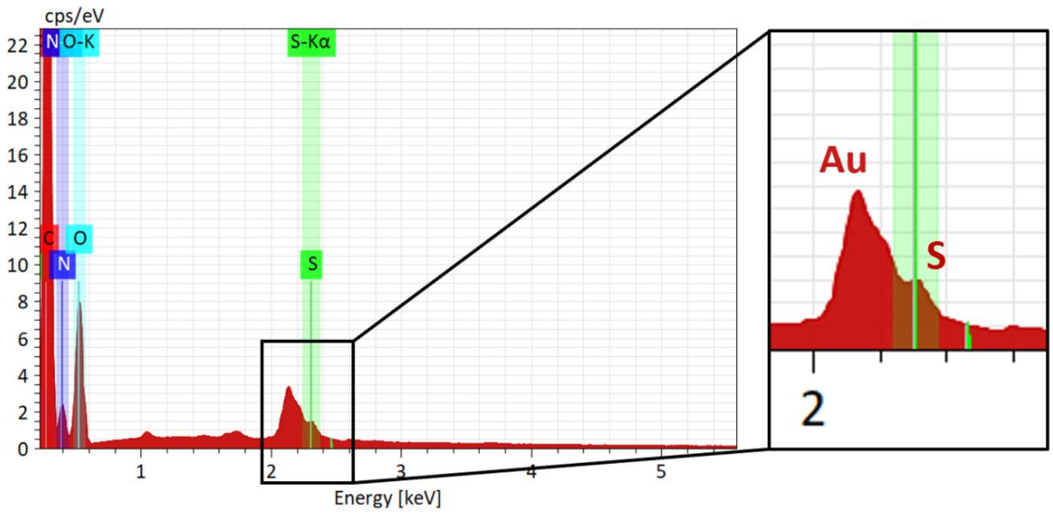

Figure S2. EDS spectrum of PGC2 (C, N, O, and S).

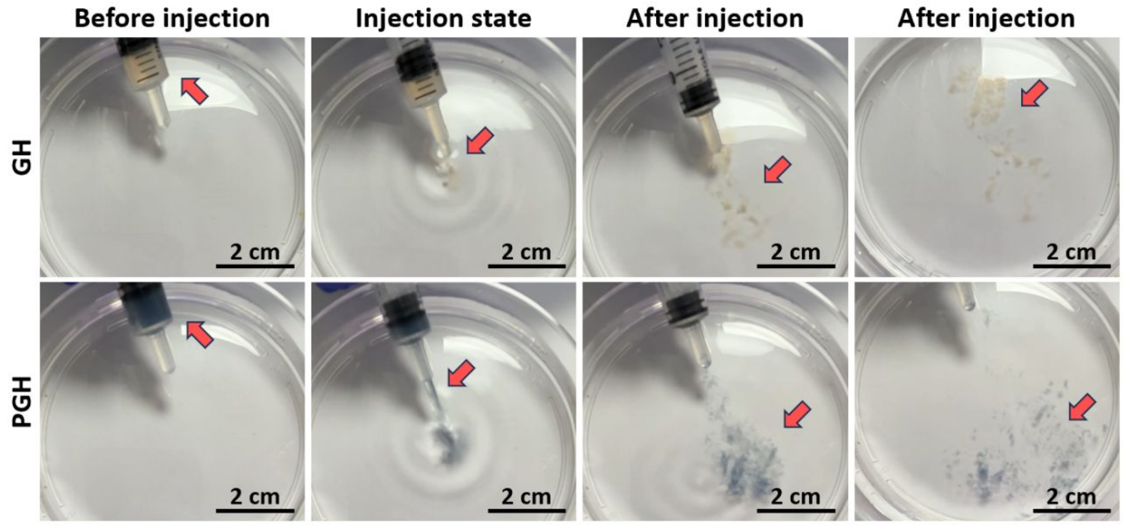

Figure S3. Photographs of the GH and PGH before and after passing through a syringe.

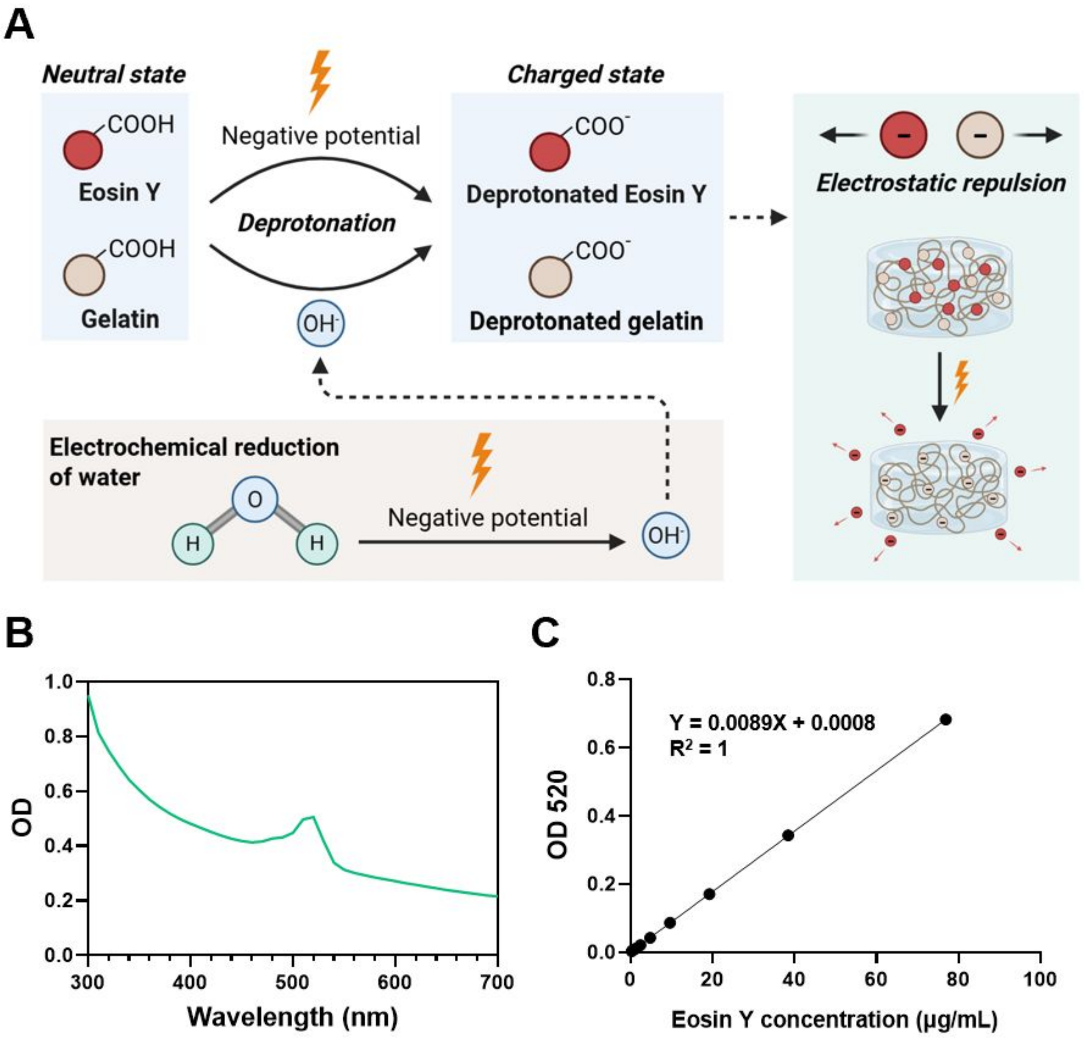

Figure S4. (A) Schematic illustration of the electrically responsive release mechanism of Eosin Y from cryogel. (B) Full-wavelength scan result of Eosin Y. (C) Standard curve of Eosin Y.

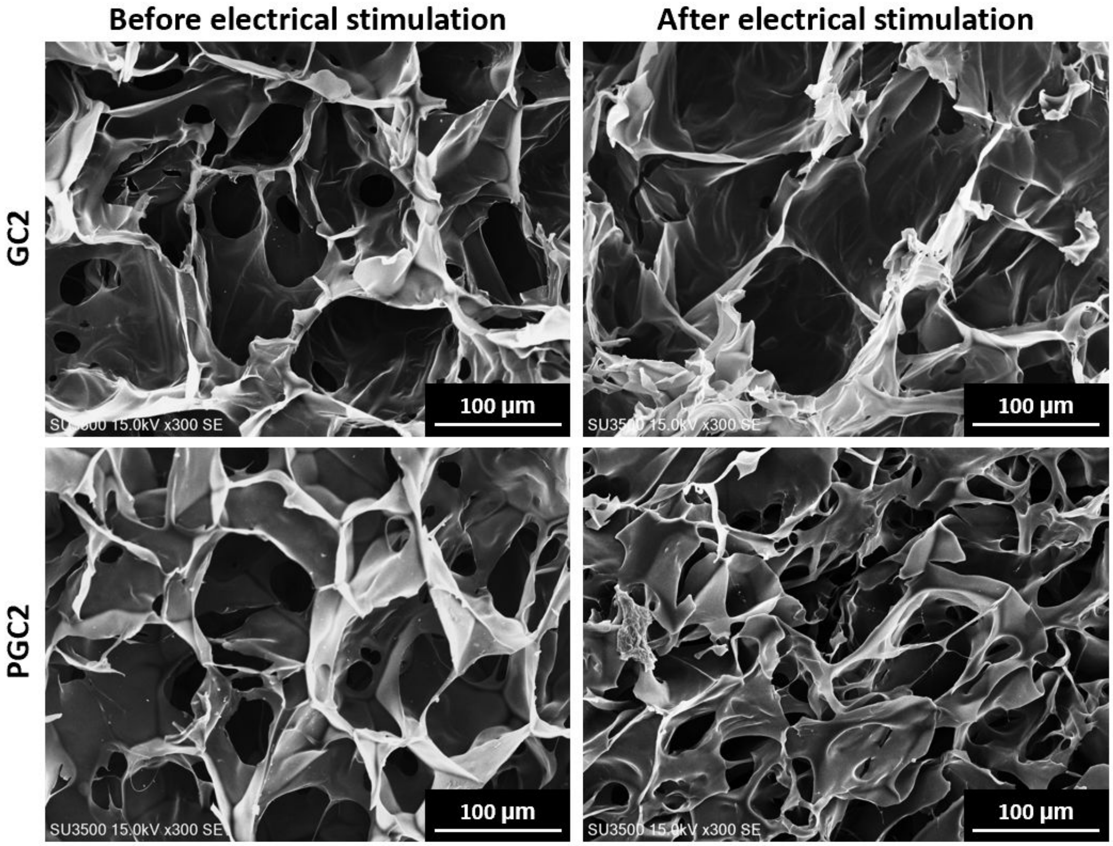

Figure S5. SEM images of GC2 and PGC2 before and after electrical stimulation (15 min).
